# The Hospital Saturday Fund

**Published:** 1913-02-15

**Authors:** Henry Robinson

**Affiliations:** London, S.W.


*To the Editor of* The Hospital.

### The Hospital Saturday Fund.

*To the Editor of* The Hospital.

Sir,—The very frank letter from Mr. G. A. Lewcock which appears in your last week's issue is both timely and interesting. Timely because of the disturbance of the old order of affairs by the Insurance Act, and because of the falling off in last year's Hospital Saturday Fund; interesting because it reveals a point of view which the voluntary hospitals cannot afford to neglect.

The points which he raises require answers, and I will take them *seriatim*. First, as to the allegations of discourtesy on the part of almoners, though this is really only incidental to the more important questions of principle. I should be disposed to scout this complaint altogether if it were brought against any of the four or five almoners with whose work and ways I have a first-hand acquaintance. But since it is made in general terms, and since all almoners may not be as tactful and judicious as the ladies I refer to, all that can be said is that the facts of each case should be laid before the governing body of the particular hospital at which the almoner's methods create dissatisfaction. If the Committee of the Saturday Fund did this, I cannot imagine that any hospital board would refuse a hearing and a careful investigation. Let me further point out that the business of an almoner is to protect the charity she serves from abuse by those who are not fit objects of its benefits; that such abuse is not uncommon; and that to achieve her end the almoner's inquiries have to be searching, though, of course, they should not be insulting. Those who present hospital letters from the Saturday Fund cannot be exempted from this process of inquiry, for the Fund has no direct interest in checking hospital abuse.

This brings me to the next question, "Are our subscribers entitled to treatment in respect of the amount subscribed by them to the hospitals?" This question does not admit of a simple yes or no, so it will be necessary to ask Mr. Lewcock's indulgence whilst a somewhat wider view of the question of hospital treatment is taken.

The voluntary hospitals are supported partly by the income from the endowments of past generations of charitable people, partly by the contributions of the charitable of to-day, and partly by the contributions of the classes who chiefly use them, through organisations of which the Hospital Saturday Fund is a type and an example. They exist to provide treatment of many different kinds for those whose circumstances are such that they cannot obtain what is necessary for their individual cases from their own resources; and to afford emergency treatment for accidents, irrespective of the pecuniary position of the victims. Thus a man or woman may be fitly admitted to hospital for some grave internal tumour, which requires the highest surgical skill for its removal, only to be obtained "in private" at an expense which may run into hundreds of pounds; while the same person may rightly be refused treatment for a cough or a boil, for which they can easily afford to pay some practitioner a fee of a few shillings. The true test is, in fact, Can this patient provide the requisite treatment for himself?—not, Has this man a recommendation from the Hospital Saturday (or any other) Fund?

But that is not the only difficulty in Mr. Lewcock's "main question." Who is to be the judge of the value of the "treatment in respect of the amount subscribed" by the Saturday Fund to the hospitals? Because the services of the medical staffs are given for nothing, does that mean that the Saturday Fund values them at nothing, and assesses the value of hospital treatment simply on the basis of the hospital expenditure per patient per day, as revealed in annual balance sheets? Does anyone suppose, too, that the only return which the Saturday Fund subscribers get is an occasional admission of a patient with a Saturday Fund letter? I think if the matter could be gone into thoroughly, it would be found that the vast majority of the subscribers and their dependants who obtain treatment from voluntary hospitals, either as out-patients or in-patients, get it easily enough without any letter at all. Because they get it without any formal intervention from the Saturday Fund, surely that is not a reason for refusing cognisance of it? I should be very glad to hear from Mr. Lewcock that he does not overlook these factors; but it is his use of the term "consideration for the purchase money" which leads me to suspect that he may have done so. The "consideration" which the subscribers to the Saturday Fund get is in fact enormously greater than the out-of-pocket cost to the hospitals of such persons as the Saturday Fund supplies with letters. It is greater, first, by the treatment accorded to the subscribers or their dependants otherwise than on presentation of letters; secondly, by the unremunerated services of the medical and surgical staffs, who in the case of general hospitals in large towns such as London are the heads of their profession; thirdly, by such items as a proportion of the interest on capital outlay on land, buildings, and equipment of all kinds, which in practically every case have been provided by the liberality of previous generations of charitable people.

This letter is, I fear, already too long; but may I present for Mr. Lewcock's reflection the problem of the future? It can be briefly stated. If Saturday Funds and similar collections break down or cease to exist, the voluntary hospitals may cease to exist too. If that happens, State intervention will be necessary. The subscriptions of the charitable will vanish, and the medical profession will no longer do the work for nothing, or next to nothing. The burden thus placed on the State will be a very heavy one indeed. Part of it will perhaps be placed direct on the wage-earner, as in the Insurance Act; and even that part which is directly borne by the State comes to a certain degree from the wage-earners. To buttress up the voluntary system by steady support is a policy which Saturday Fund subscribers will find infinitely cheaper than any form of State intervention. There are many other reasons for the preservation of that system (in the interests of the patients), but they are not now stated, inasmuch as Mr. Lewcock's argument is devoted to the commercial side of the question.

Finally, it is true that by custom many hospitals issue "subscriber's letters" of recommendation at some specified rate of letters to subscription. But it is not true that the hospitals in so doing are selling, or that the subscribers in question are "purchasing," that number of rights to admission; or that the amount of subscription which entitles a subscriber to one letter represents the (average) value of the treatment of one patient, nor even a small fraction of it. If every subscriber demanded his pound of flesh in like manner, every voluntary hospital which issues letters would be bankrupt in a year. It is bad news that there is dissatisfaction among Hospital Saturday subscribers; but it would seem to be based, at least to some extent, upon a misconception of the functions and duties of the hospitals and of their financial relations to the organised small subscriber.—I am, Sir, yours faithfully,

Henry Robinson.

London, S.W., February 9, 1913.

